# Parameter estimation without confidence intervals?

**DOI:** 10.4178/epih.e2016036r

**Published:** 2016-08-19

**Authors:** Toru Fukuhara, Yusuke Hori

**Affiliations:** Department of Neurological Surgery, National Hospital Organization Okayama Medical Center, Okayama, Japan

We greatly appreciate the comments contributed by one of the peer reviewers. Apparently, his comments have clearly sorted out the limitations our article involves. We recognize all the limitations and still, we believe the Diagnosis Procedure Combination (DPC) system in Japan is worth being aware of as a novel medical database in Asia.

As for the first point, the DPC database only involves 65.1% of general beds, so most readers may think it insufficient for calculating the incidence, even as an assumption. It is difficult for us to describe the statistical characteristics of the 34.9% non-DPC general beds, but those are mostly inactive, and the management of newly diagnosed spontaneous intracerebral hemorrhage (sICH) without referring to the DPC hospitals must be quite minor. Considering the acute and severe onset of sICH, most sICH cases are captured in the DPC hospitals. The masked rate, noted as the second issue, has been troublesome for the prefectural analysis, but the number we lost during analysis is calculable, at around 10%. We believe this limitation can be avoidable in the near future by accessing this database directly as a certified researcher, since the qualification process for analyzing the whole content of this database is currently being established. Then, how can we prove the adequacy of the calculated incidence from defective case numbers? This is mentioned as the third limitation, and the correspondent insists that it be concluded that the DPC data is worthless for the estimation of the incidence. We feel the statistical results we presented are marginal, but also difficult to dismiss as meaningless. The correspondent may be right; however, the value of the DPC system as a medical database should be recognized because of its attractive features, which we explained in the article.

The main objective of our article is presenting the profile of this database system, and the presentation of the statistics may be insufficient, including lacking confidence intervals, partly because the word limits of the journal. Instead, all the data for the statistics are available in the Appendix; thus the analysis can be replicated. We, the authors, hope that many readers of this journal find value in our article.

